# Synthesis of indium oxide microparticles using aerosol assisted chemical vapour deposition†

**DOI:** 10.1039/d0ra02678f

**Published:** 2020-06-11

**Authors:** Firoz Alam, David J. Lewis

**Affiliations:** Department of Materials, The University of Manchester Oxford Road Manchester M13 9PL UK david.lewis-4@manchester.ac.uk; Department of Chemistry, The University of Manchester Oxford Road Manchester M13 9PL UK

## Abstract

Microparticles of indium oxide (In_2_O_3_) are deposited on glass substrates at 500 °C using aerosol assisted chemical vapour deposition (AACVD). The structural, morphological and optical properties of the as-deposited particles are reported.

## Abstract 2

Microparticles of indium oxide (In_2_O_3_) are deposited on glass substrates at 500 °C using aerosol assisted chemical vapour deposition (AACVD).

## Graphical abstract

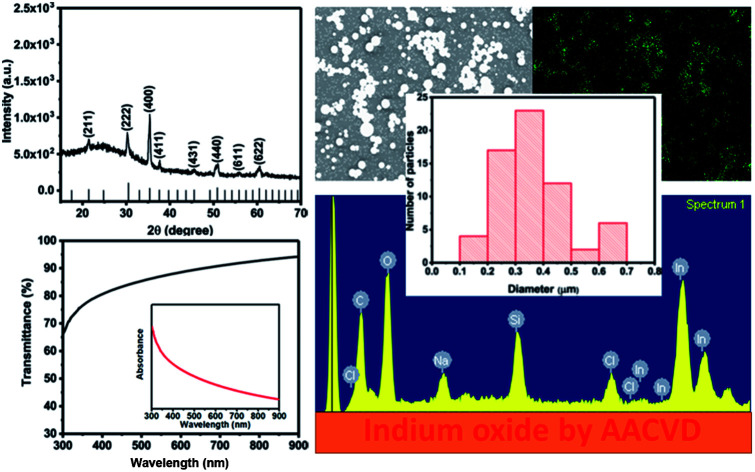

Indium oxide (In_2_O_3_) is a promising wide bandgap n-type semiconductor with good optical transparency in the visible region and has drawn significant interest in the field of photovoltaics,^1,2^ thin film transistors,^3^ photodetectors^4^ and gas sensors.^5^ Doping with other metals (such as Sn, Mo, Zr, Ga, Ti and Ta) is possible in order to modify the properties of In_2_O_3_, whilst retaining optical transparency in the visible region of the electromagnetic spectrum. Examples of these widely used doped materials include (i) tin-doped indium oxide (ITO), which is a well known transparent conducting oxide (TCO) has an optical bandgap of 3.4 eV, good chemical stability and excellent adhesion to the substrate, widely used in optoelectronic devices such as light emitting diodes^6^ and solar cells;^7^ (ii) recently, molybdenum-doped In_2_O_3_ (IMO) has been reported as an durable alternative to the commercially dominant ITO.^8^ IMO has shown higher conductivity and increased infrared transparency than ITO with the same carrier density. This makes IMO a more suitable and low cost alternative material for device applications as much thinner films of IMO can produced with properties equal to or better than ITO of the same carrier concentration; (iii) Zr-doped indium oxide, which has been used as a transparent electrode in perovskite/silicon tandem devices, which results in the improvement of the power conversion efficiency from 23.3% to 26.2%;^1^ and (iv) Ga-doped indium oxide which has been used in phase change memory devices.^9^

Several deposition techniques have been used to produce In_2_O_3_ thin films such as atomic layer deposition,^10^ molecular beam epitaxy,^11^ pulsed laser deposition,^12^ spin coating,^2^ metal organic chemical vapour deposition^13^ and aerosol assisted chemical vapour deposition.^14,15^ The latter technique is useful as it is simple, low cost, single step and used in the industry for assembly line glass coating. Maeng *et al.* reported In_2_O_3_ films using Et_2_InN(TMS)_2_ as a liquid precursor and H_2_O in the temperature range of 225–250 °C.^10^ Kim *et al.* has used spin coating to deposit In_2_O_3_ using indium chloride as precursor and annealed at 400 °C.^3^ In both the cases a poorly crystalline XRD pattern is reported. Basharat *et al.* has reported the deposition of In_2_O_3_ film at 550 °C on glass substrate from the dual-source AACVD reaction of Me_3_In and ROH (R = CH_2_CH_2_NMe_2_, CH(CH_3_)CH_2_NMe_2_, C(CH_3_)_2_CH_2_OMe, CH_2_CH_2_OMe) in toluene.^14^ The chosen ligands are less air/moisture sensitive and have increased solubility, but the preparation of precursor ligands are time consuming (24 h reaction), multi-stepped and require low temperatures (−78 °C). Yang *et al.* reported a one-step aqueous solvothermal method for the synthesis of highly crystalline and nearly monodisperse In_2_O_3_ nanocrystals.^16^ Seo *et al.* reported the preparation of colloidal, highly crystalline and size controlled In_2_O_3_ nanoparticles from thermal decomposition of the In(acac)_3_ precursor in oleylamine.^17^ These latter methods yield highly crystalline and monodisperse nanoparticles, but are quite time consuming and not suitable for mass production of In_2_O_3_. A comparison table of AACVD and other reported methods for producing indium oxide has been incorporated into the ESI.† The structural phase stability, optical properties, electronic structure and high pressure behaviour of In_2_O_3_ has been studied by Karazhanov *et al.* using first-principles density functional theory (DFT) calculations in three different space group symmetries *I*2_1_3, *Ia*3̄ and *R*3̄. It is found that In_2_O_3_ with space group *Ia*3̄ undergoes a pressure-induced phase transition to the R3 phase at *ca.* 3.8 GPa. For In_2_O_3_ the magnitudes of the absorption and reflection coefficients with these space group symmetries are small and in the energy range of 0–5 eV, indicating that these phases are transparent.^18^

In this paper we report the deposition of transparent indium oxide microparticles on glass substrates using aerosol assisted chemical vapour deposition (AACVD) from a single precursor solution. AACVD is an ambient pressure CVD technique which is simple, cost-effective, proceeds in a single step and is suitable for the production of large area thin films on a range of substrates, and has been used for the deposition of wide range of semiconducting materials such as MAPBr,^19^ Cs_2_SnI_6_,^20^ MoS_2_,^21^ Cr-doped MoS_2_,^22^ SnS^23^ and copper zinc tin sulfide (CZTS).^24^ Solubility of the precursor molecules in a solvent is required in order to obtain high quality thin films. A number of indium precursors have been used to deposit high quality In_2_O_3_ thin films and whilst current precursor design is functional, none of them are entirely satisfactory and have certain disadvantages,^14^ for example, [In-(OCMe(CF_3_)_2_)_3_(H_2_N^*t*^Bu)] and [Me_2_In(OC(CF_3_)_2_CH_2_-NHMe)] contain fluorine, which results in fluorine contamination in In_2_O_3_ films.^25,26^ [In(thd)_3_] (thd = 2,2,6,6-tetramethylheptane-3,5-dionate) was synthesised and added to dichloromethane (CH_2_Cl_2_), toluene and tetrahydrofuran (THF) separately, but in all cases a fine suspension is formed which, upon standing for 1 h, is sedimented.^15^ Indium halides are known for their poor solubility in non-coordinating organic solvents and often rapidly disproportionate to In^II^ or In^III^ halide complexes with treatment of coordinating solvents.^27^ Keeping the solubility of precursor in mind, we have used for the first time a mixture of polar aprotic solvents to completely dissolve InI to obtain a clear transparent precursor solution for the deposition of In_2_O_3_ on glass substrates using AACVD.

The precursor solution was obtained by dissolving indium iodide (InI) powder in a mixture of anhydrous *N*,*N*-dimethylformamide (DMF) and acetonitrile (1 : 1, v/v) with stirring for 1 h at 70 °C. Aerosols were generated from this solution using an ultrasonic humidifier, which were then transported using a stream of argon gas (250 sccm) into a hot wall reactor containing cleaned glass substrates at a temperature of 500 °C. After decomposition of the precursor a transparent film comprised of In_2_O_3_ microparticles is obtained. The as-deposited materials were characterised using powder X-ray diffraction (p-XRD) in the range of 10° < 2*θ* < 70° (Fig. 1). Reflections from the (211), (222), (400), (411), (431), (440), (611) and (622) Bragg planes of cubic In_2_O_3_ were observed at 2*θ* = 21.49°, 30.58°, 35.46°, 37.68°, 45.69°, 51.03°, 55.99° and 60.67° respectively (ICCD no. 00-006-0416, space group *Ia*3̄ with *a* = 10.11 Å). The reflection indexed to the (400) plane is dominant, which indicates a preferred orientation of growth along this direction under these conditions.^8^

**Fig. 1:**
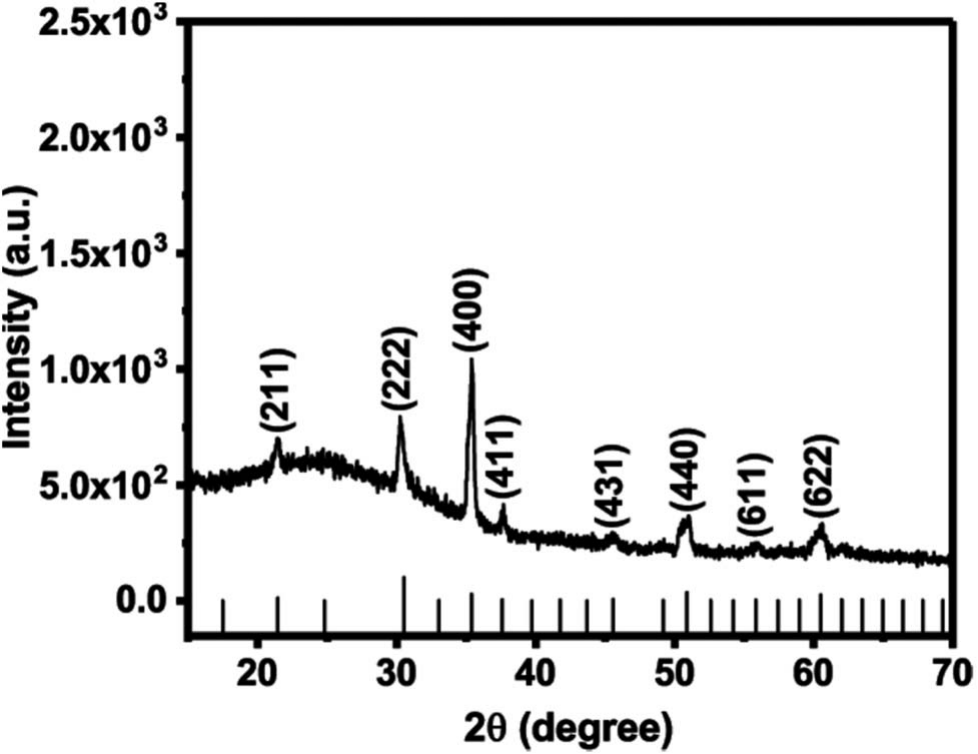
PXRD pattern of In_2_O_3_ microparticles deposited by AACVD on glass substrates at 500 °C. The black sticks represent the theoretical powder diffraction pattern of cubic In_2_O_3_ (ICCD file no. 00-006-0416).

The Raman spectrum of the In_2_O_3_ microparticle film was collected using 514 nm laser excitation. Scattering peaks at 130, 551 and 1371 cm^−1^ were observed (Fig. 2). The peak around 130 cm^−1^ is assigned to the A^(1)^_g_ vibrational mode and peak at 551 and 1371 cm^−1^ are ascribed to the phonon vibrational modes of cubic In_2_O_3_. These values are consistent with those reported in literature.^28–30^ The remaining peaks around 1100 and 1600 cm^−1^ are assigned to carbon which may arise from decomposition of solvent.^31^

**Fig. 2:**
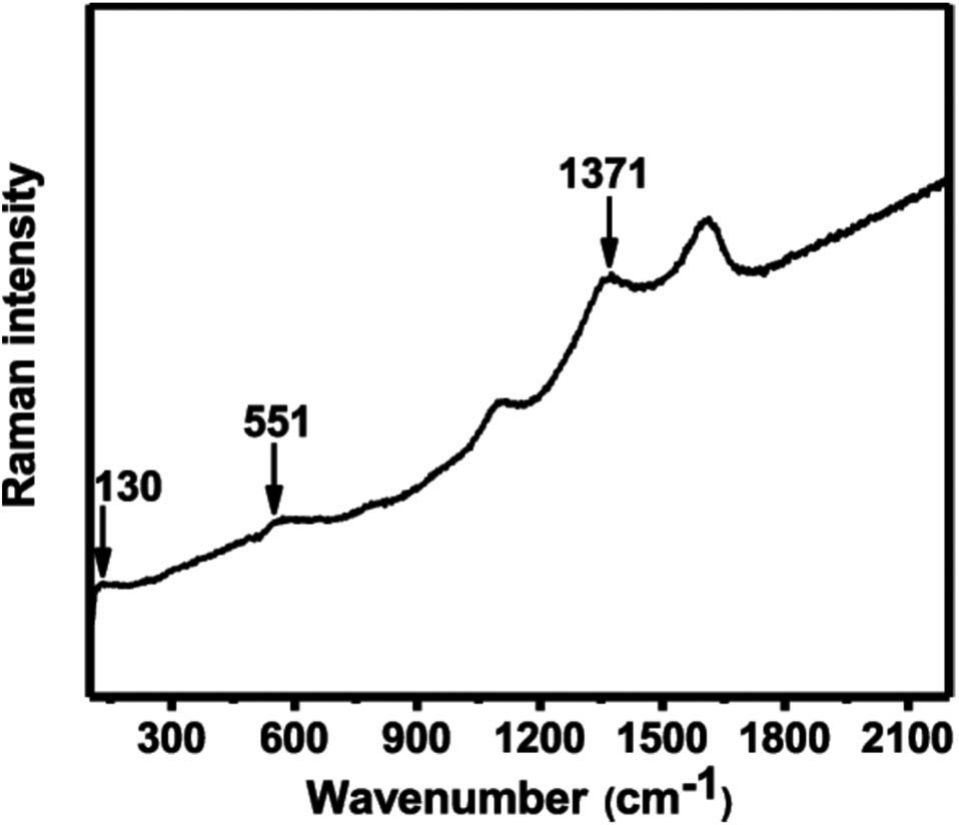
Raman spectrum of In_2_O_3_ microparticles deposited by AACVD on a glass substrate at 500 °C.

The surface morphology of the as-deposited material was investigated using scanning electron microscopy (SEM) in secondary electron mode. Spherical microparticles are uniformly and randomly distributed over the substrate (Fig. 3(a)). Fig. 3(b) shows the distribution of indium (In) over an interrogated area of *ca.* 15 × 11 μm confirmed by energy dispersive X-ray (EDX) mapping at an acceleration voltage of 10 kV. Fig. 3(c) shows a histogram which summarises the size distribution of In_2_O_3_ (*N* = 65). The average diameter of an In_2_O_3_ particle is 368 ± 120 nm. The mechanisms of indium oxide particle growth and resulting particle sizes have been discussed in literature. Maensiri *et al.* reported that the average particle size of In_2_O_3_ increases with increasing the calcination temperature.^32^ Ayeshamariam *et al.* reported that crystallite sizes derived from XRD increases with increasing annealing temperature and that the band gap energy of these particles also scales linearly as a function of annealing temperature. It was not expected to be a quantum confinement effect as the particle sizes studied (*ca.* 30 nm) were all an order of magnitude greater than the exciton Bohr radius of In_2_O_3_ (*ca.* 3 nm).^33^ Elemental composition of our In_2_O_3_ microparticle film was performed using EDX. The EDX spectrum and atomic percent of the elements present in In_2_O_3_ film are shown in Fig. S1 and S2,† giving a clear indication of the presence of In in the film.

**Fig. 3:**
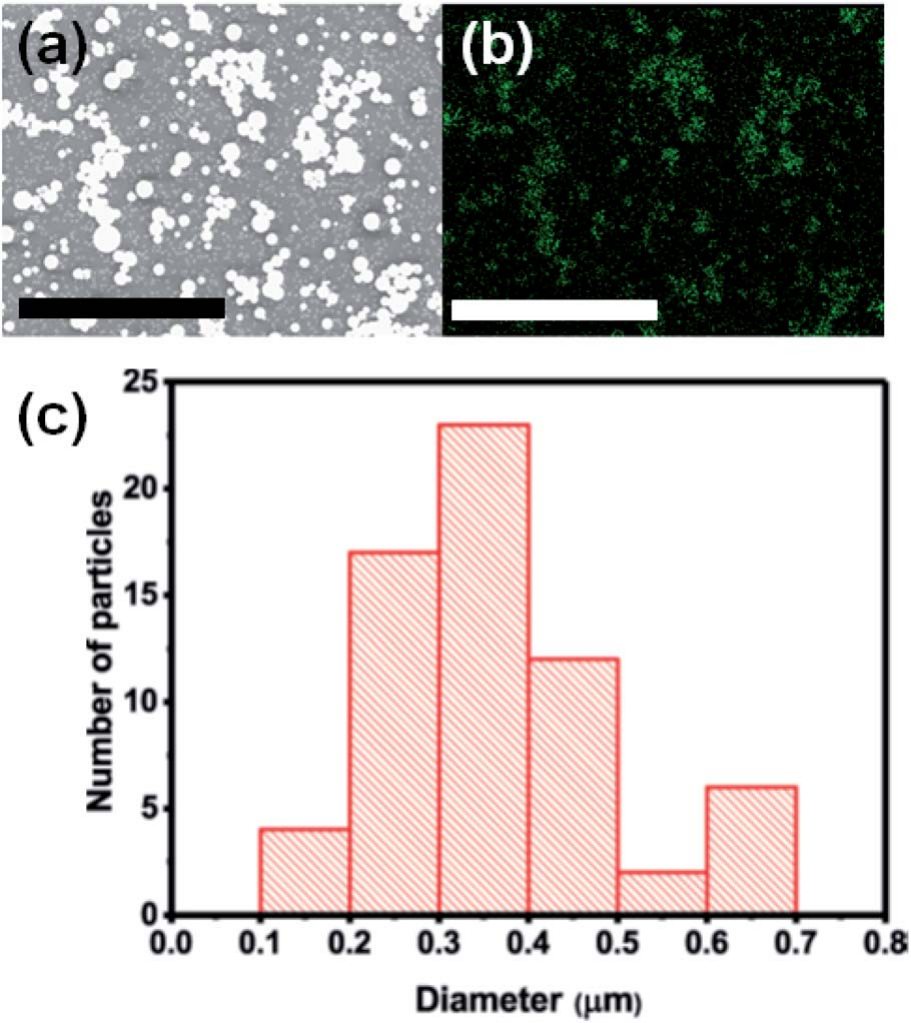
Characterisation at the microscale using electron microscopy. (a) Secondary electron (SE) SEM image, (b) spatially resolved EDX spectrum mapping of In emission over the same area and (c) histogram of In_2_O_3_ microparticles deposited by AACVD on a glass substrate at 500 °C (*N* = 65). The scale bar in (a) and (b) corresponds to 7 μm in each case.

The optical properties of as-deposited thin film of In_2_O_3_ microparticle was investigated by UV-Vis-NIR transmittance in the wavelength range 300–900 nm. The as-deposited In_2_O_3_ shows over 80% transmittance in the wavelength range of 400–900 nm (Fig. 4), which is comparable to previous reports.^10^ The inset of Fig. 4 shows an absorbance spectrum of In_2_O_3_ microparticles revealing strong optical absorbance in the ultraviolet region of the spectrum. Estimation of optical bandgap of In_2_O_3_ microparticle thin film from Tauc plot (described in full in the ESI Section S5†) gives a direct band gap value of 3.53 eV (Fig. S3†). Given the band gap value of 3.53 eV, the In_2_O_3_ produced by this method and under these conditions is thus a wide band gap semiconductor. By visual inspection it is observed that the In_2_O_3_ film deposited on glass substrate is colourless and transparent to visible light.^3,34^

**Fig. 4:**
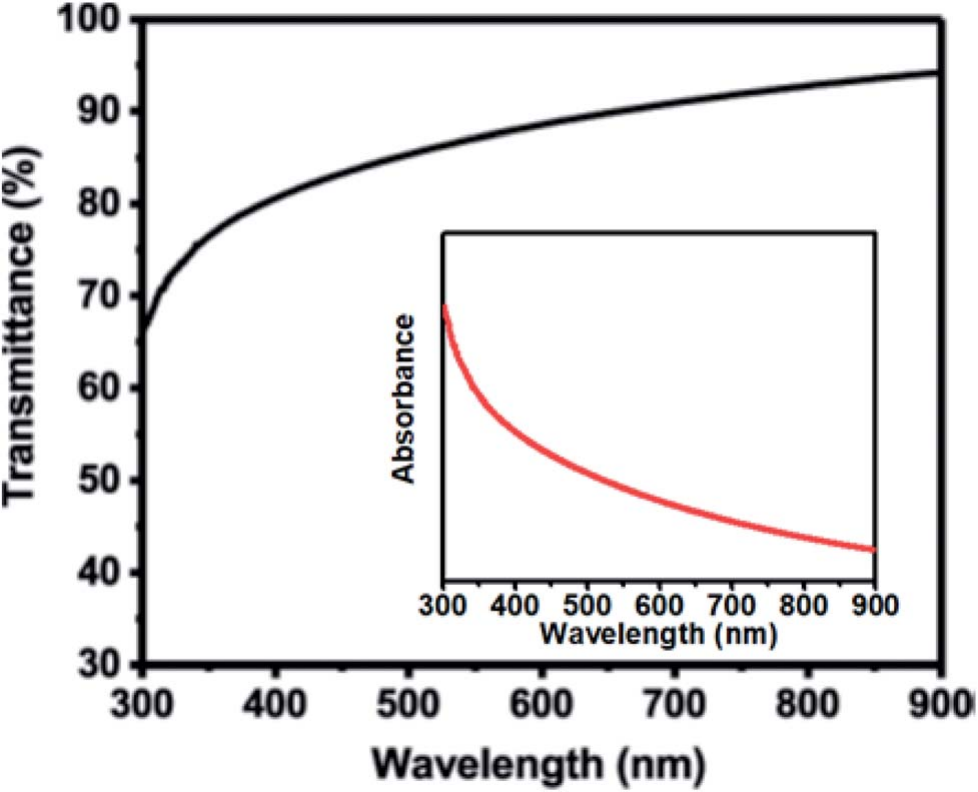
Optical transmittance spectrum of In_2_O_3_ microparticles deposited by AACVD technique on a glass substrate at 500 °C. Inset: absorbance spectrum of as-deposited In_2_O_3_ microparticles.

In summary, a simple, cost-effective, and single step aerosol assisted chemical vapour deposition (AACVD) technique has been used for the synthesis of optically transparent spherical In_2_O_3_ microparticles on glass substrates. Powder XRD patterns and Raman scattering confirm the crystallinity of as-deposited In_2_O_3_ microparticles with a preferred orientation along the (400) plane. Inspection of surface morphology by secondary electron SEM shows that the as-deposited In_2_O_3_ microparticles are spherical with an average diameter of 368 ± 120 nm (*N* = 65) and are uniformly and randomly distributed over the substrate. EDX spectroscopy gives a clear indication of the presence of In on the substrate. The UV-Vis-NIR spectroscopy of as-deposited In_2_O_3_ microparticle film show over 80% transmittance in the wavelength range of 400–900 nm and strong optical absorbance in the ultraviolet region of the spectrum with an estimated direct bandgap value of 3.53 eV from a Tauc plot. Optical data thus suggest that bandgap value of 3.53 eV does not fall in the band gap range for semiconductors, and hence materials produced by this method are wide band gap semiconductors. Thus the AACVD method we report produces indium oxide from a soluble carbon-free precursor with potential for scale-up.

## Conflicts of interest

The authors declare no conflicts of interest.

## Supplementary Material

RA-010-D0RA02678F-s001
