# Supplementary material for: Synthesis of indium oxide microparticles using aerosol assisted chemical vapour deposition
Source: RSC Adv. 2020 Jun 11;10(38):22487–90. doi: 10.1039/d0ra02678f (PMC9054642; doi:10.1039/d0ra02678f)
Supplement: RA-010-D0RA02678F-s001 [file RA-010-D0RA02678F-s001.pdf]

Electronic Supplementary Material (ESI) for RSC Advances.
This journal is © The Royal Society of Chemistry 2020

Electronic Supporting Information (ESI)

Synthesis of Indium Oxide Microparticles using Aerosol Assisted Chemical Vapour Deposition

Firoz Alam [ab] and David J Lewis *[a]

[a]Department of Materials, The University of Manchester, Oxford Road, Manchester, M13 9PL, United Kingdom. E-mail: david.lewis-4@manchester.ac.uk
[b]Department of Chemistry, The University of Manchester, Oxford Road, Manchester, M13 9PL, United Kingdom

S1. Materials

Solvents were purchased from Merck and Sigma-Aldrich and used without further purification. Indium iodide (InI) was purchased from Sigma-Aldrich.

S2. Instrumentation

Powder X-ray diffraction (p-XRD) pattern of thin film deposited on glass substrate was measured using a Bruker AXS D8 Discover diffractometer, using copper Kα radiation (λ = 1.54178 Å). Films was scanned over the range of 2θ = 10 - 70˚ using a step size of 0.02 ˚ with a dwell time of 0.5 s.

Raman spectroscopy was performed using a Renishaw system 1000 Raman spectrometer equipped with a long focal length 50x objective. The Raman spectrum was acquired using the WiRE 1.3 software using a laser with an excitation wavelength of 514 nm (25% power) and spectrometer configuration: (Diffraction Grating: 1800 lines/mm, extended mode, accum: 3, gain: high, Cosmic ray: off and charge coupled device (CCD) area detector, calibrated to the 520 nm with silicon as reference). Spectrum presented here is a raw data, no background subtraction and smoothing has been performed.

Scanning electron microscope (SEM) analysis was performed using a Zeiss Ultra 55 FEG-SEM + EBSD + EDX in secondary electron mode with an accelerating voltage of 10 KV. Energy-dispersive X-ray (EDX) analysis was carried out using the same instrument with an accelerating voltage of 10 KV using an Oxford Instruments INCA pentaFETx3 detector. Carbon coating on the film was carried out using an Edwards E306A coating unit. The thin film for SEM and EDX analysis was mounted on stub using copper tape and earthed with silver paint.

UV-Vis-NIR transmittance and absorption spectrum of thin film was recorded using a Shimadzu UV-1800 instrument over a range of 300-900 nm.

S3. Precursor solution

A clear and transparent carbon-free precursor solution is obtained by dissolving 250 mg of InI powder to 5 mL of mixed solvent of N,N-dimethyl formamide and acetonitrile (1:1, v/v) with stirring at 550 r.p.m for 1 hour at 70 ˚C.

S4. Aerosol-assisted chemical vapour deposition of In_2O_3 thin film

A transparent thin films In_2O_3 were deposited on cleaned glass substrates (1.5 cm x 3 cm) using aerosol-assisted chemical vapour deposition technique from a clear solution mentioned above in section *S3*. The apparatus used for AACVD in the laboratory has been previously described by Ramasamy et al. (Ramasamy, *Chem. Mater.*, 2013, **25**, 266-276). The flow rate of the argon carrier gas was 250 sccm and the tube furnace temperature was set to 500 ˚C. The AACVD run until all the precursor solution is finished. The In_2O_3 thin films were taken out from the tube furnace further analysis, once the temperature is reached below 100 ˚C.

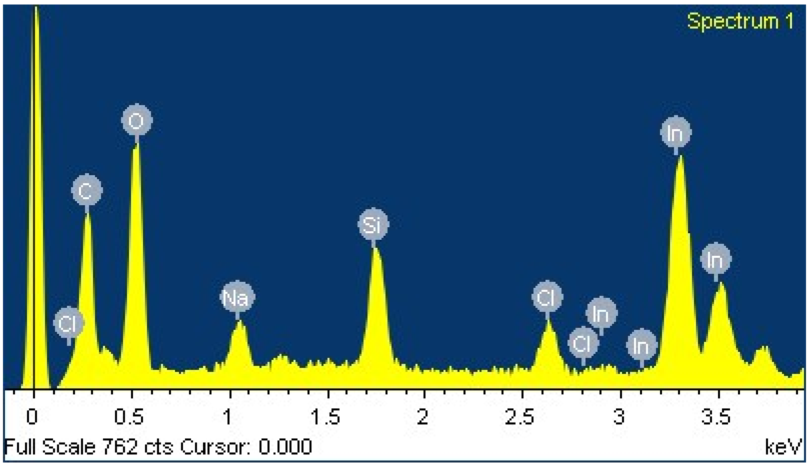

Element	Weight%	Atomic%
C K	8.63	22.85
O K	25.52	50.71
Na K	1.96	2.71
Si K	4.71	5.33
Cl K	3.24	2.91
In L	55.94	15.49
Totals	100.00	

Fig. S1 shows the EDX plot (left) and atomic percent of the elements present in the film (right) of In_2O_3 microparticle thin film deposited by AACVD technique on glass substrate at 500 ˚C.

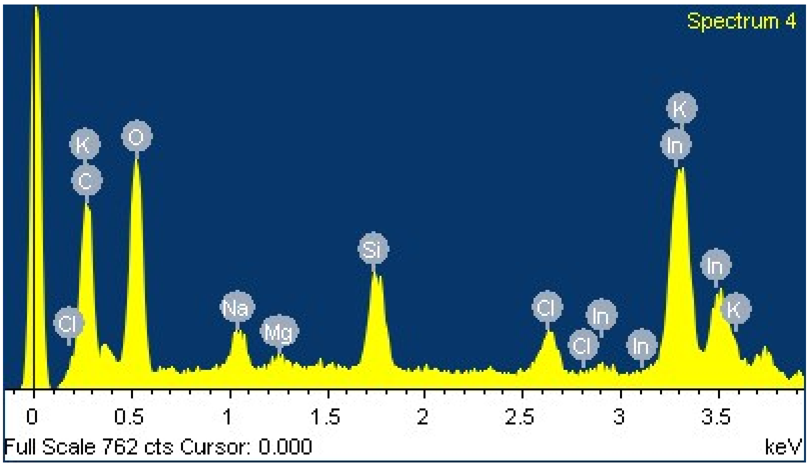

Element	Weight%	Atomic%
C K	7.24	19.40
O K	26.36	53.01
Na K	1.77	2.48
Mg K	0.33	0.44
Si K	4.10	4.69
Cl K	3.03	2.75
K K	2.23	1.84
In L	54.94	15.40
Totals	100.00	

Fig. S2 shows the EDX plot (left) and atomic percent of the elements present in the film (right) of In_2O_3 microparticle thin film deposited by AACVD technique on glass substrate at 500 ˚C.

S5. Optical bandgap calculation using Tauc plot

Optical bandgap of In_2O_3 microparticle thin film has been determined using Tauc plot ($(\alpha h\nu)^2$ vs $h\nu$) obtained from the absorbance data of In_2O_3 microparticle thin film deposited by AACVD technique on glass substrate at 500 ˚C. The estimated bandgap is determined by extrapolation of the linear region to energy axis intercept, indicating a direct bandgap value of 3.53 eV, which is consistent with previously reported literature values.[1,2]

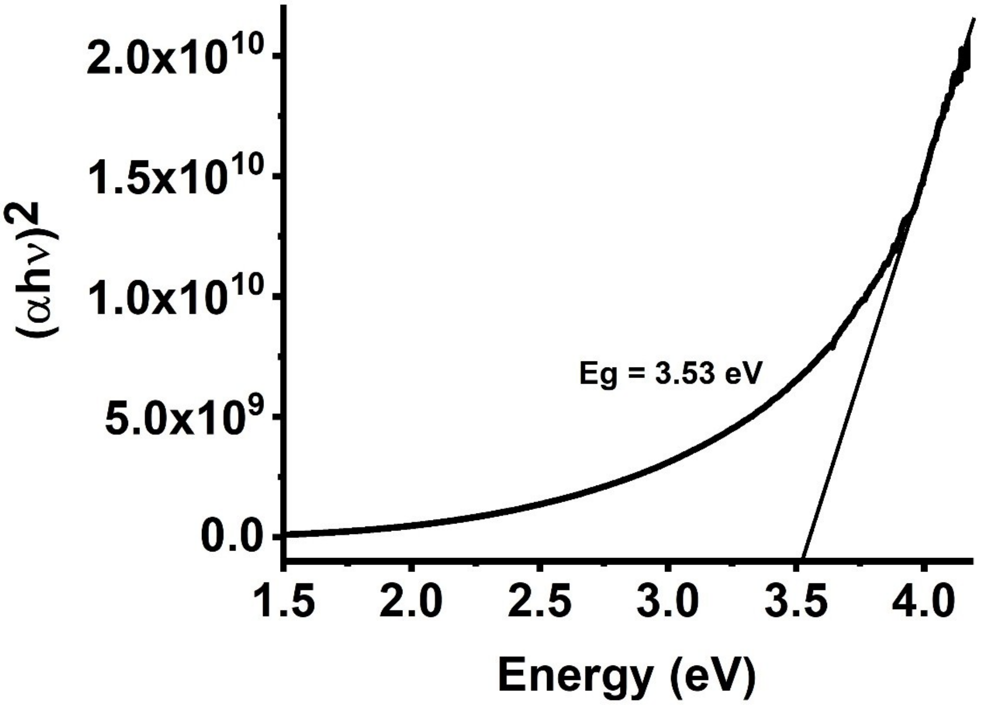

Fig. S3 shows the Tauc plot obtained from the absorbance data of In_2O_3 microparticle thin film deposited by AACVD technique on glass substrate at 500 ˚C.

Table 1 shows the comparison of advantages/efficiency of present method over the reported methods for producing indium oxide.

Methods	Advantages	disadvantages	Ref.
Aerosol assisted chemical vapour deposition	Work on ambient pressure, simple, cost-effective, proceeds in a single step and has been adapted by industry for assembly-line glass coating (e.g. Pilkington) and is suitable for the production of large area thin films on a range of substrates.		[3]
Spin Coating	Widely used due to its simplicity, quickly and easily produce small area films, low cost, ease of set up.	Impossible to scale up because it is extremely difficult to obtain uniform spin coated films over a large glass substrate, reproducibility is very less and its two step technique, wastage of materials, only allows one substrate at a time.	[4]
Thermal oxidation	Slow oxidation rate, good control of the oxide thickness.	Time consuming and not suitable for mass production.	[5]
Chemical vapour deposition	High growth rate and reproducibility, good quality uniform films over large area, batch processing for higher throughput, low fabrication cost.	Requirement of high vacuum systems makes them less attractive, High capital cost.	[6, 8]
Atomic layer deposition	High quality films, conformality, uniformity, low-temperature processing, stoichiometric control, multilayer, excellent repeatability.	Economic viability, high material waste rate, high energy waste rate, nanoparticle emissions, deposition rate slower than CVD.	[7,8]
Solvothermal	Highly crystalline and nearly monodisperse In_2O_3 nanocrystals.	Expensive, large amount of solvent waste generated, very time consuming and multistep processing to get nanocrystals	[9]
Thermal decomposition	Monodisperse colloidal nanoparticles, highly crystalline and size controlled In_2O_3 nanoparticles.	Very time consuming, require multistep such washing few times using centrifuge and not suitable mass production.	[10]
Molecular beam epitaxy	Flexibility in growing various compounds and structures with precise doping and compositional profiles accurate to atomic dimensions, epitaxial growth rate can be varied from 0.1 µm/hr to 10 µm/hr, precise control the layer thickness.	Required an ultra-high vacuum environment, very expensive and complicated system, Asaro–Tiller–Grinfeld (ATG) instability.	[11,12]

References

1. P. D. C. King, T. D. Veal, F. Fuchs, C. Y. Wang, D. J. Payne, A. Bourlange, H. Zhang, G. R. Bell, V. Cimalla, O. Ambacher, R. G. Egdell, F. Bechstedt, and C. F. McConville, *Phys. Rev. B*, 2009, **79**, 205211-10.
2. A. Murali, A. Barve, V. J. Leppert and S. H. Risbud, *Nano Lett.*, 2001, **1**, 287-289.
3. D. J. Lewis and P. O'Brien, *Chem. Commun.*, 2014, **50**, 6319-6321.
4. H. S. Kim, P. D. Byrne, A. Facchetti and T. J. Marks, *J. Am. Chem. Soc.*, 2008, **130**, 12580-12581.
5. K. Soulantica, L. Erades, M. Sauvan, F. Senocq, A. Maisonnat and B. Chaudret, *Adv. Funct. Mater.*, 2003, **13**, 553-557.
6. C. Y. Wang, V. Cimalla, H. Romanus, T. Kups, G. Ecke, T. Stauden, M. Ali, V. Lebedev, J. Pezoldt and O. Ambacher, *Appl. Phys. Lett.*, 2006, **89**, 011904.
7. W. J. Maeng, D.-w. Choi, K.-B. Chung, W. Koh, G.-Y. Kim, S.-Y. Choi and J.-S. Park, *ACS Appl. Mater. Interfaces*, 2014, **6**, 17481-17488.

8. P. O. Oviroh, R. Akbarzadeh, D. Pan, R. A. M. Coetzee and T.-C. Jena, *Sci. Technol. Adv. Mater.*, 2019, **20**, 465-496.
9. J. Yang, C. Li, Z. Quan, D. Kong, X. Zhang, P. Yang and J. Lin, *Cryst. Growth Des.*, 2008, **8**, 695-699.
10. W. S. Seo, H. H. Jo, K. Lee and J. T. Park, *Adv. Funct. Mater.*, 2003, **15**, 795-797.
11. O. Bierwagen, M. E. White, M.-Y. Tsai and J. S. Speck, *Appl. Phys. Lett.*, 2009, **95**, 262105-262107.
12. A. Y. Cho, Integrated Circuits: Chemical and Physical Processing, 1985, *ACS Symposium Series*, **290**, 118-126**.**
